# Impact of the Lorentz force on electron track structure and early DNA damage yields in magnetic resonance-guided radiotherapy

**DOI:** 10.1038/s41598-022-18138-3

**Published:** 2022-09-30

**Authors:** Yoshie Yachi, Takeshi Kai, Yusuke Matsuya, Yuho Hirata, Yuji Yoshii, Hiroyuki Date

**Affiliations:** 1 Graduate School of Health Sciences, Hokkaido University, Kita-12 Nishi-5, Kita-ku, Sapporo, Hokkaido 060-0812 Japan; 2 Nuclear Science and Engineering Centre, Research Group for Radiation Transport Analysis, Japan Atomic Energy Agency (JAEA), 2-4 Shirakata, Tokai, Ibaraki 319-1195 Japan; 3 Central Institute of Isotope Science, Hokkaido University, Kita-15 Nishi-7, Kita-ku, Sapporo, Hokkaido 060-0815 Japan; 4 Faculty of Health Sciences, Hokkaido University, Kita-12 Nishi-5, Kita-ku, Sapporo, Hokkaido 060-0812 Japan

**Keywords:** Biological physics, Cell death, Medical research

## Abstract

Magnetic resonance-guided radiotherapy (MRgRT) has been developed and installed in recent decades for external radiotherapy in several clinical facilities. Lorentz forces modulate dose distribution by charged particles in MRgRT; however, the impact of Lorentz forces on low-energy electron track structure and early DNA damage induction remain unclear. In this study, we estimated features of electron track structure and biological effects in a static magnetic field (SMF) using a general-purpose Monte Carlo code, particle and heavy ion transport code system (PHITS) that enables us to simulate low-energy electrons down to 1 meV by track-structure mode. The macroscopic dose distributions by electrons above approximately 300 keV initial energy in liquid water are changed by both perpendicular and parallel SMFs against the incident direction, indicating that the Lorentz force plays an important role in calculating dose within tumours. Meanwhile, DNA damage estimation based on the spatial patterns of atomic interactions indicates that the initial yield of DNA double-strand breaks (DSBs) is independent of the SMF intensity. The DSB induction is predominantly attributed to the secondary electrons below a few tens of eV, of which energy deposition patterns are not considerably affected by the Lorentz force. Our simulation study suggests that treatment planning for MRgRT can be made with consideration of only changed dose distribution.

## Introduction

Magnetic resonance-guided radiotherapy (MRgRT) has been developed to achieve high tumour control probability (TCP) with suppressed side effects by virtue of real-time imaging of soft tissues with high contrast^1,2^. In recent decades, MRgRT employing photon beams, such as linear accelerated X-rays or ^60^Co γ-rays, in static magnetic fields (SMF) for MR imaging^1–3^ has been installed in several clinics. When treating tumours with the MRgRT system, electron beams as well as secondary electrons generated by photons can be affected by the Lorentz force in transverse SMF against incident beams, leading to dose enhancement, the so called electron return effect (ERE)^4^. To date, some biological experiments have shown enhanced radio-sensitivities (e.g., chromosome aberration and cell death) even at the same dose level in magnetic fields^5–8^, whereas others have suggested that radio-sensitivities of X-ray irradiation are unaffected by magnetic fields^9–11^. Because the experimental results in the literature do not show consistent results, the radio-sensitivity under magnetic fields remains uncertain. In order to clarify the radiosensitivity, it is necessary to evaluate the relationship between radiation track structure and biological impacts.

To investigate the impact of magnetic fields on radiation-induced biological effects based on the radiation track structure, a Monte Carlo computational simulation for radiation transport is a powerful approach. There are several Monte Carlo codes for simulating electron track structure developed worldwide^12–14^. Amongst the codes, PENELOPE^15,16^, Geant4-DNA^17^ and TOPAS-nBio^18^ have provided micro- and nano-dosimetric quantities in magnetic fields, showing the relationship between energy deposition and the biological impact in SMFs^19,20^. These simulations have suggested no significant enhancement of dose deposition at the DNA scale in SMFs^19,20^. Meanwhile, to our knowledge, there is no report estimating the various types of DNA damage yields, i.e., double-strand breaks (DSBs) and other complex forms. To investigate the mechanisms on DNA damage induction in SMFs, so it is of importance to estimate early DNA damage yields utilizing the first-principles method.

For dealing with the above issues, Particle and Heavy Ion Transport code System (PHITS)^21^ is appropriate because the dynamics of low-energy electrons down to 10^–3^ eV in liquid water^22,23^ can be analysed using the electron track-structure mode, *etsmode*, even in magnetic fields, where the types and the yields of DNA damage are determined based on the spatial patterns of atomic interactions^24,25^. In this study, we estimate the physical features of electron tracks (i.e., ranges, dose distributions, etc.) in SMFs and the early DNA damage yields through physical process simulations. This work finally shows that treatment planning for MRgRT can be made considering both change of dose distribution and unaffected biological impacts.

## Materials and methods

### Simulation setup for electron transport in SMF

The PHITS code version 3.27^21^ was used for simulating electron tracks in liquid water. In the PHITS simulations, the [track-structure] section was activated within liquid water, which enables us to calculate each atomic interaction (i.e., elastic scattering, ionization, electronic excitation, dissociative electron attachment, vibrational excitation, rotational excitation and phonon excitation) along an electron track based on *etsmode*^25,26^. To consider the Lorentz force in the PHITS simulation, we also used the [magnetic field] section for electrons.

### Electron track structure analysis and calculation of physical quantities

Dose distributions by 10-MeV electrons parallel to the SMF along the x- and y-axes, and 1-MeV electrons perpendicular to the SMF along the x- and z-axes were calculated by the electron gamma shower (EGS)^27^ mode in PHITS, which is a condensed-history approach for simulating electron kinetics for energies down to 1 keV. The two types of ranges, i.e., penetration length and projected range, were also calculated. It should be noted that the penetration length is defined as the length of the vector from the point of departure to the final position of the electron after thermalization, whereas the projected range is the length between the departure and the final position projected to the axis in the incident direction. These ranges of electrons with monoenergetic energy from 100 to 1000 keV in various SMF intensities were calculated.

To verify the simulation accuracy of *etsmode* in the SMF, we compared the result of PHITS *etsmode* with that of EGS. The cut-off energies for *etsmode* and EGS were 1.0 eV and 1.0 keV, respectively. In addition, we simulated the electron tracks in vacuum for checking the electron trajectory changed by the SMF (without electron scattering by the interaction with liquid water). Since PHITS estimates the radiation track from the corresponding mean-free path without considering a constant time interval (i.e., 1 attosecond), we adopted a time-dependent variational Monte Carlo method, dynamic Monte Carlo code (DMCC)^22,23^. The physical model for simulating electron dynamics by DMCC was implemented in *etsmode*, thus the validation using DMCC can be applied to the calculation results using *etsmode*. From the DMCC simulation, we obtained the gyration time and radius of the electrons in one period within the SMF. Various electron energies (0.01–1000 keV) were also simulated and compared with the projected range for 50–1000 keV electrons. Both physical quantities were calculated with large numbers of electrons to make the uncertainties small less than a few percent in general.

### Estimation of DNA damage yields

To evaluate the impact of magnetic fields on DNA damage yields, we used an analytical code for estimating DNA damage implemented in PHITS version 3.27. In the DNA damage simulation, the energy deposition and the density of the inelastic events (i.e., ionization and electronic excitation) calculated by PHITS *etsmode* were scored. In detail, assuming that ionization and electronic excitation are potential causes to induce DNA strand breaks, we scored the number of the event pairs (so-called linkage) within 3.4 nm per track *N*_link_. Note that two strand breaks within 3.4 nm (corresponding to 10 bp) is regarded as a DSB. Assuming that the number of linkages per track *N*_link_ per energy deposition *E*_dep_ is proportional to the DSB induction, the DSB yield *Y*_DSB_ is given by

1 $$Y_{{{\text{DSB}}}} { = }k_{{{\text{DSB}}}} \frac{{N_{{{\text{link}}}} }}{{E_{{{\text{dep}}}} }}$$

where *k*_DSB_ is the proportionality constant (keV/Gy/Da), which was found to reproduce the experimental yields of DSB after exposure to 220 kVp X-rays^25^. Note that the DNA damage estimation model has been in good agreement with experimental data and other simulations in the previous study^25^, which was also verified in this study as shown in Fig. 4. Based on Eq. (1), we obtained the *Y*_DSB_ values for 0.1, 1.0, 10, 100, 300 keV electrons in SMF intensities *B* from 0.0 to 10.0 (T). To quantitatively evaluate the secondary electron impact, we also estimated the *Y*_DSB_ value when not considering the generation of secondary and Auger electrons. In addition, we estimated the fraction of clustered forms, related with complex DSBs, based on a previous modelling^24^. In this model^24^, 12 events (i.e., ionizations and electronic excitations) are needed on average for inducing an additional strand break at a DSB site. Based on the model, the DSB complexity was estimated by the number of the events (*N*_cl_) within a sampling site of 10 bp radius (i.e., DSB site), namely 2 ≤ *N*_cl_ < 14 for simple DSB, 14 ≤ *N*_cl_ < 26 for DSB+ , and 26 ≤ *N*_cl_ < 38 for DSB+ + . Note that DSB+ is the DSB coupled with a SB within 10 bp, and DSB+ + is the DSB coupled with two SBs within 10 bp^28^. This simple cluster model has been well benchmarked from the comparison with experimental data using atomic force microscopy (AFM)^24^. The DNA damage simulation was performed by tracking a large number of electrons to make uncertainties small sufficiently.

## Results and discussion

### Electron dose distribution in magnetic fields

**Figure 1:**
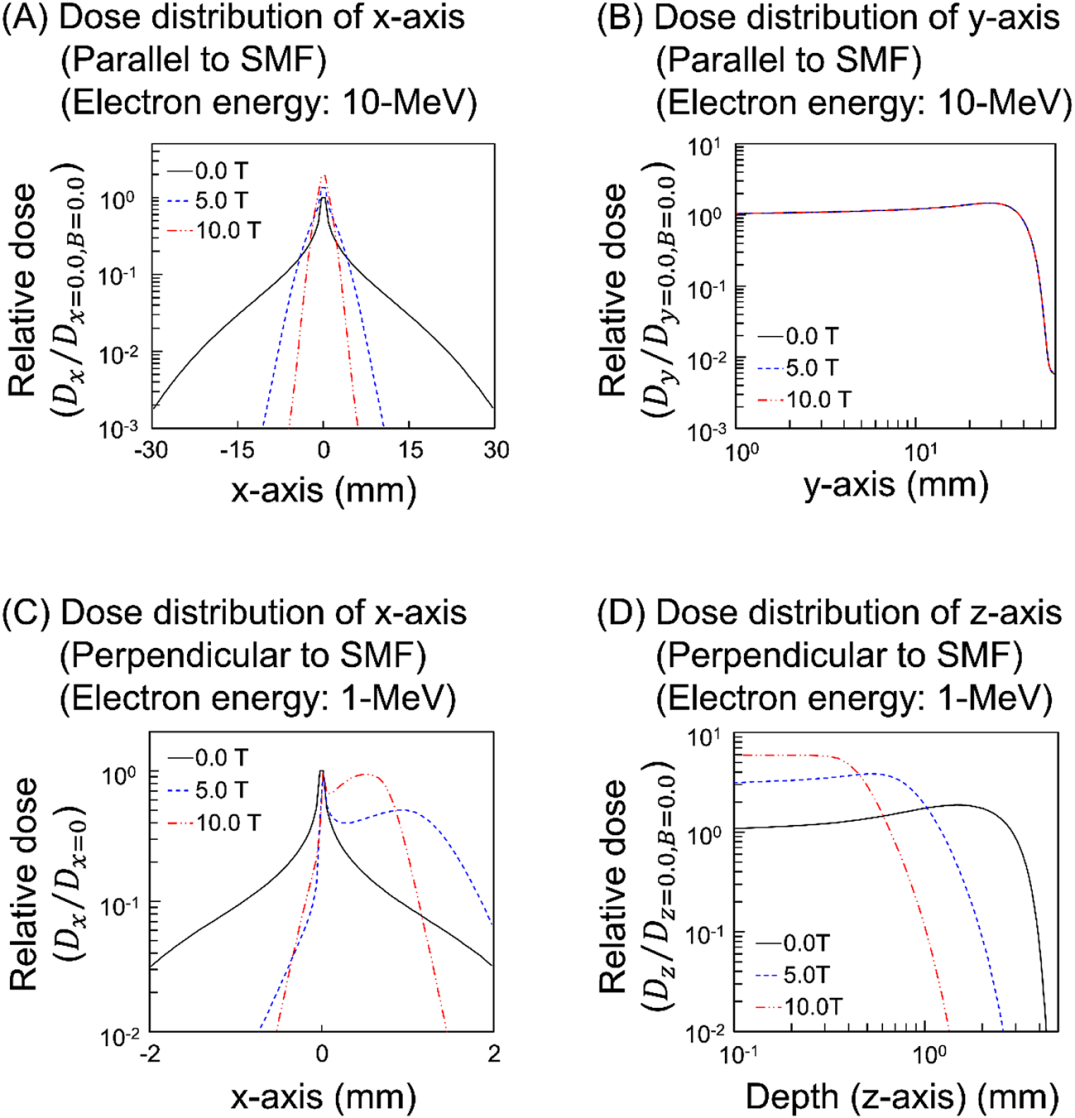
Dose distribution of electrons in SMFs. Dose distributions along the x-axis (**A**) and y-axis (**B**) for 10-MeV incident electrons in the positive y-axis direction parallel to the SMF calculated by EGS. The distributions along the x-axis (**C**) and z-axis (**D**) for 1-MeV incident electrons in the positive z-axis direction perpendicular to the SMF calculated by EGS. We confirmed that the distributions calculated by *etsmode* were in good agreement with those by EGS as shown in supplementary data (Fig. S2). The distributions for 10-MeV and 1-MeV electrons were significantly affected by the SMFs.

The dose distributions for 10-MeV electrons parallel to SMFs (*B* = 0.0, 5.0, 10.0 T) are shown in Fig. 1A and B. In the case of the field parallel to the incident electron direction, the larger the SMF strength, the narrower to the x- or z-axis the electron beams is (Fig. 1A). The depth-dependency of dose (y-axis) is independent of the SMF (Fig. 1B). Meanwhile, the dose distributions for 1-MeV electrons perpendicular to SMFs (*B* = 0.0, 5.0, 10.0 T) are shown in Fig. 1C and D. The dose distribution without a SMF is symmetrical with respect to x = 0. However, those perpendicular to SMFs are largely biased in a large SMF strength (Fig. 1C). Focusing on depth-dependencies perpendicular to the SMF, as the SMF becomes larger, the energies are deposited over less depth (Fig. 1D). The change of dose distributions is analogous to the modification of track structures confirmed in supplementary data (see Fig. S1). Note that the distributions calculated by *etsmode* were confirmed in supplementary data (see Fig. S2) and these results were in good agreement with the calculated results by EGS.

The validity of EGS mode has been checked in the report on the PHITS benchmark results^29^, in which the dose distribution of 10-MeV electrons calculated by the EGS mode was in good agreement with the experimental data. The dose distribution calculated by *estmode* is shown in Figs. S2 and S3, where the result by *estmode* agrees well with that by the EGS mode and the measured dose distribution (see supplementary material). The results by the PHITS simulation show that the trajectories of 10-MeV and 1-MeV electrons, which are used in radiation therapy, are significantly affected by magnetic fields. However, we confirmed that the dose distributions by low-energy electrons are not affected by the SMF as shown in supplementary data (see Fig. S4).

### Impact of magnetic fields on projected ranges of electrons

**Figure 2:**
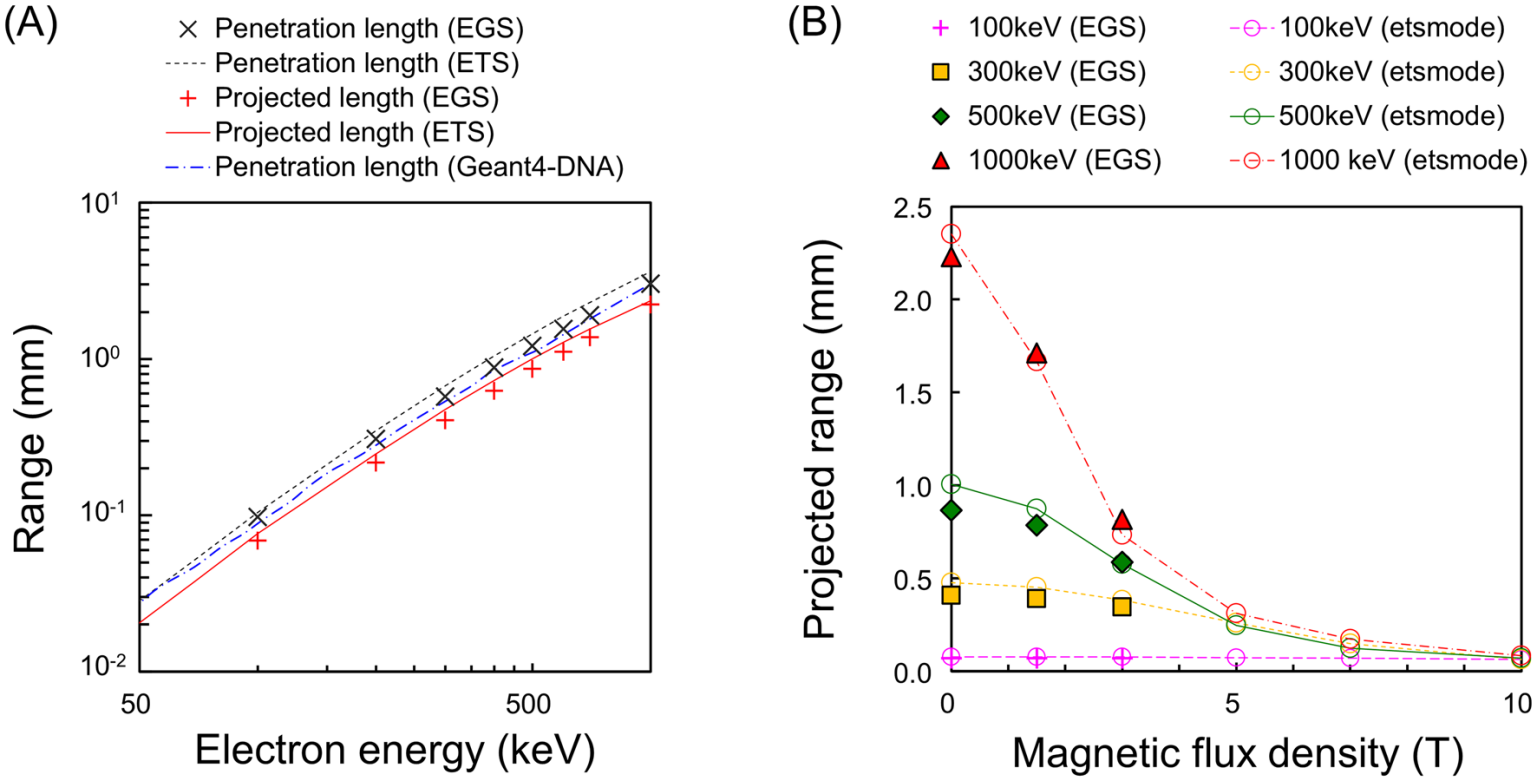
Electron ranges in SMFs calculated by *etsmode* and EGS. (**A**) shows the penetration length and projected range in the absence of a SMF (*B* = 0.0 T) and (**B**) shows the projected range in the presence of various SMF strengths (*B* = 0.0–10.0 T). The simulation results by *etsmode* and EGS are in good agreement with each other and other simulation results by the Geant4-DNA toolkit^30^. As shown in Fig. 2B, the projected range of high-energy electrons is largely affected by the SMF strength.

Figure 2 shows the ranges of electrons perpendicular to the SMF as a function of incident electron energy. To verify the accuracy of electron track structure in the SMF simulated by *etsmode*, we compared the electron ranges calculated by *etsmode* to those by EGS. The simulation accuracy of *estmode* in the absence of a SMF has been extensively discussed in comparison with the recommended values of ICRU reports and experimental data reported previously^25^. This simulation result of range was compared with that by another simulation code, Geant4-DNA in supplementary data (see Fig. 2). As shown in Fig. 2, we compared the range given by *etsmode* to that by EGS, further affirming the accuracy of *etsmode* even in magnetic fields. Note that the difference between dose distributions by EGS mode and *etsmode* was arising from the difference of the physical model, i.e., the condensed-history method for EGS mode and the event-by-event track-structure simulation of each atomic interaction for *etsmode*. Since development of the track-structure mode in PHITS is still ongoing, the further examination will be needed to improve the simulation accuracy by comparing the result with the experimental data.

The calculated ranges for monoenergetic electrons in the absence of a SMF (*B* = 0.0 T) are shown in Fig. 2A, where the incident energy range was set to be 50–1000 keV because significant SMF effects can be expected at these energies. The projected range for monoenergetic electrons (100–1000 keV) as a function of the SMF intensity (*B* = 0.0–10.0 T) are also shown in Fig. 2B, in which there are no significant SMF effects on projected ranges for 100-keV electrons. Meanwhile, in the case of electrons with high energies above 300 keV in the presence of SMF intensity over 3.0 T, the larger the SMF strength becomes, the shorter the projected range is. These simulation results suggest that the travelling lengths along the z-direction are shortened due to the Lorenz force within the SMF, and the macroscopic dose distributions can be modified by the SMF and projected range in the SMF is shorter compared to that in without SMF.

Regarding high-energy electron beams used in radiation therapy, electrons with incident energy over 1 MeV in the presence of SMF locally deposit their energy in the region closer to their starting points, as compared to the absence of SMF (see Fig. 1D). It was therefore confirmed that the SMF effects (so called electron return effect (ERE)) for electrons in liquid water largely depend on the electron energy^19^. Also, the simulation affirms that the dose distribution within solid tumours and normal tissues should be calculated in consideration of magnetic fields when making treatment planning^2^.

### Estimation of electron return effects in vacuum

**Figure 3:**
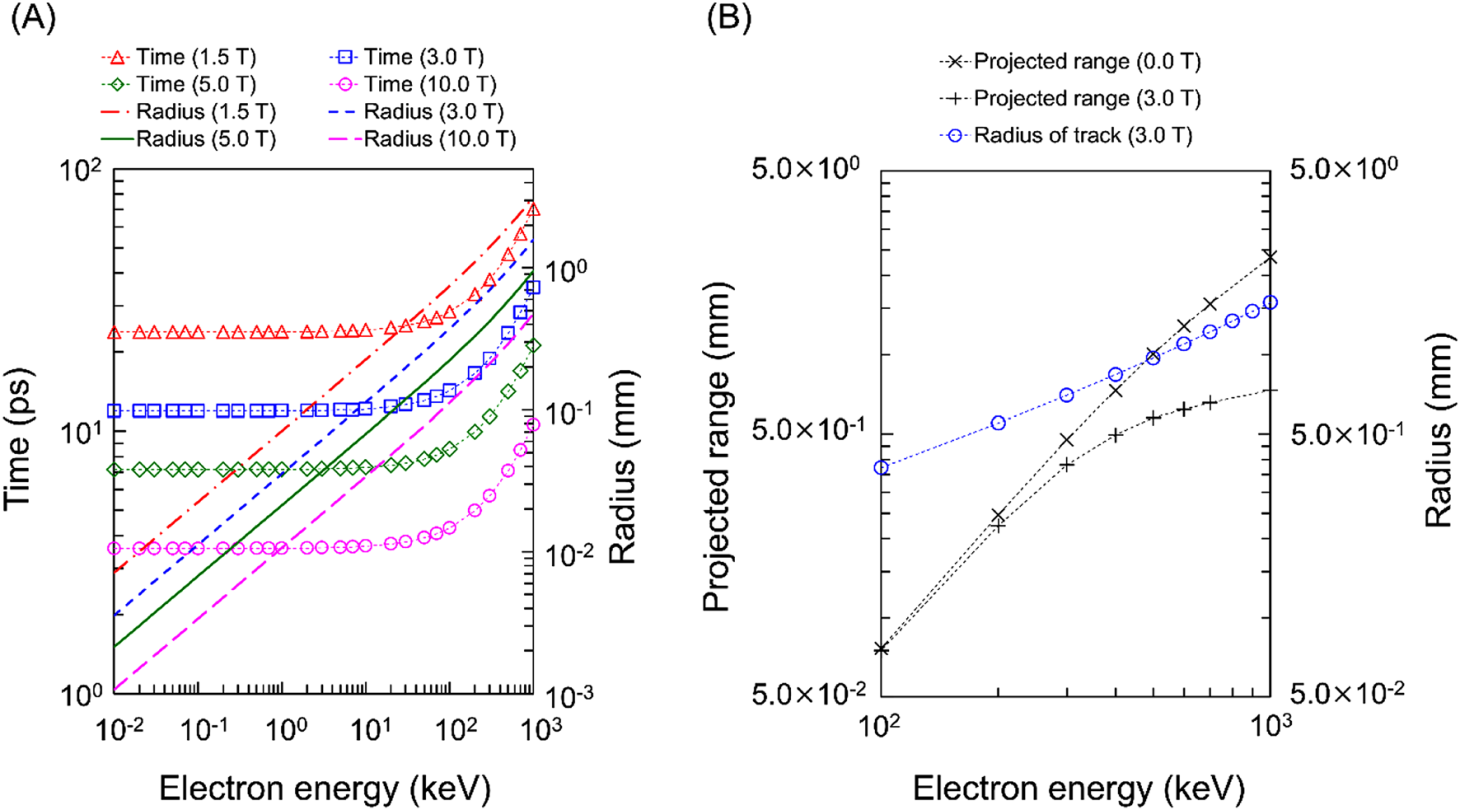
Gyration time and radius of electron tracks in vacuum in magnetic fields. (**A**) is the gyration time and the radius of monoenergetic electrons in vacuum as a function of incident electron energy. (**B**) is the gyration radius in vacuum and the projected range in liquid water for electrons with energy from 10^2^ to 10^3^ keV. In Fig. 3A, the monoenergetic electrons were simulated in various SMF strength (*B* = 0.0–10.0 T). In Fig. 3B, the radius in vacuum with magnetic fields (blue circle) was compared to the range in liquid water without magnetic fields (cross symbols) and that in *B* = 3.0 T (plus symbols). The results show that the range for electrons below a few hundreds of keV is sufficiently smaller than the gyration radius in vacuum, suggesting the impact of Lorentz force is not significant.

The gyration time and radius of electrons in vacuum in the presence of a SMF (*B* = 0.0–10.0 T) was evaluated using the DMCC^22,23^. These calculations assume that the electrons are in vacuum without considering atomic interactions. Figure 3A shows the relationship among incident electron energies, gyration time and radii. The time is constant up to approximately 100 keV, then exponentially increases above 100 keV. The radius also exponentially increases in the energy range from 0.01 to 1000 keV. In a previous study^19^, the gyration radii for electrons (0.001–100 MeV) in vacuum applied by magnetic fields were compared with the continuous slowing down approximation (CSDA) range. This result shows that electrons with energy above 100 keV, in which the CSDA range is longer than the gyration radius, are modified by the SMF (below 10.0 T). In contrast, we show the relationship between the gyration time and the radii of low-energy electrons in vacuum (Fig. 3A). It was confirmed that the flight distance and time until attenuation due to the interaction with water is shorter than the radius and gyration time for electrons with energies below a few hundreds of keV. From this relation, it was found that the electrons below a few hundreds of keV slow down before they drift by SMF (i.e., below several tens of psec).

Figure 3B shows the comparisons of projected range and the gyration radii under *B* = 3.0 (T) in liquid water and in vacuum. The projected range under *B* = 0.0 (T) is also depicted in Fig. 3B. In the case of high-energy electrons (100–1000 keV), the gyration radius monotonically increases from 0.37 to 1.58 mm as the electron kinetic energy gets higher. The radius becomes closer to the projected range. In the case of low-energy electrons (0.01–90 keV), the radius is significantly larger compared to the projected length. Therefore, high energy electrons drift in the presence of a SMF. From the relations shown in Fig. 3, it was further confirmed that the electrons with higher kinetic energy than a few hundreds of keV can be strongly affected by the SMF.

### DNA damage yields for monoenergetic electrons in a SMF

**Figure 4:**
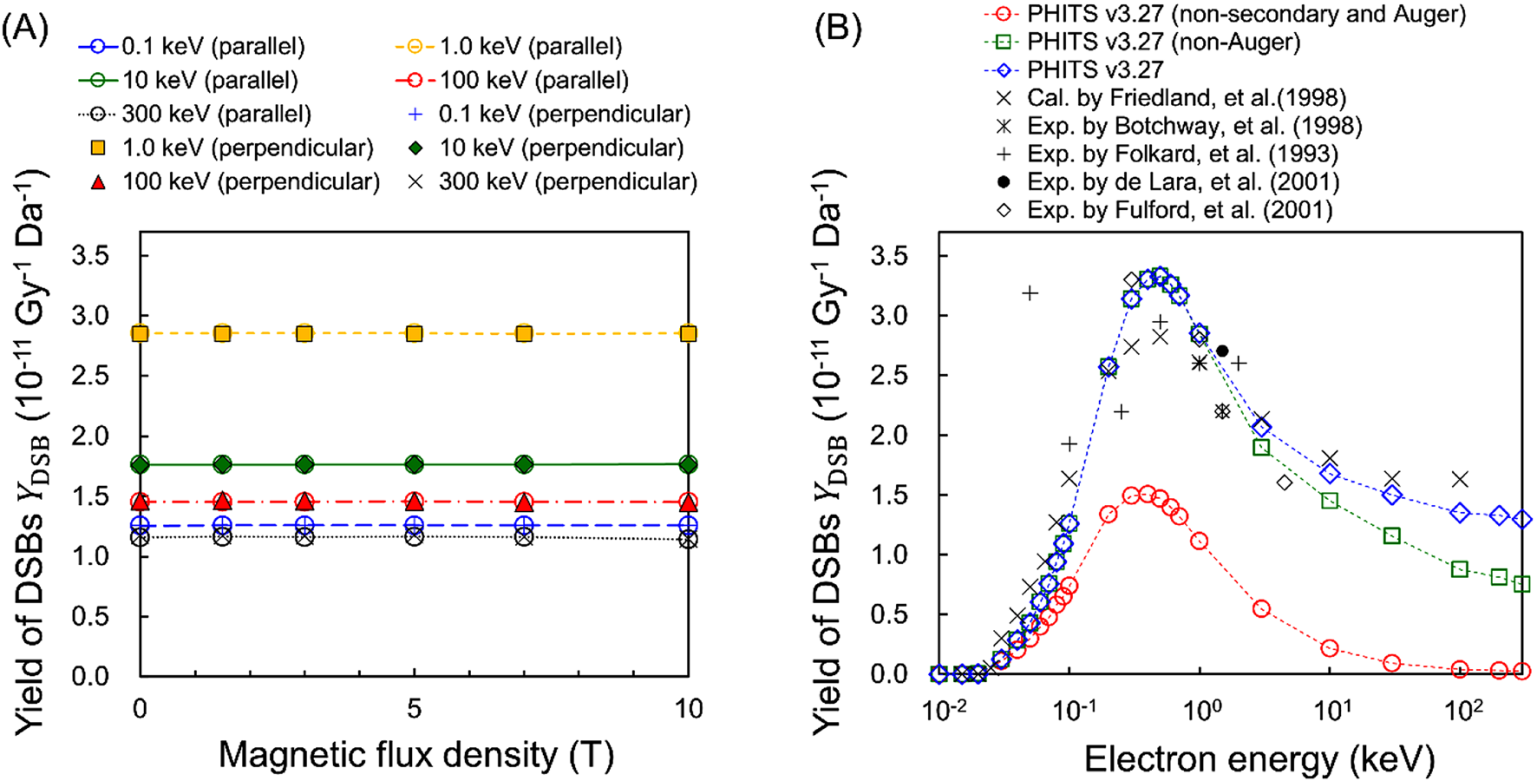
Yields of DSBs for monoenergetic electrons in SMFs. (**A**) shows the *Y*_DSB_ values for mono-energetic electrons with 0.1–300 keV electrons in the SMFs (*B* = 0.0–10.0 (T)). (**B**) shows the *Y*_DSB_ values without considering secondary electrons, including Auger electrons. The *Y*_DSB_ values were calculated using PHITS (v. 3.27) and an analytical code for estimating DNA damage yields^25^. These results are compared with other calculation data^31^ and experimental data^32–35^.

The DSB yield, *Y*_DSB_, by monoenergetic electrons in the SMFs (*B* = 0.0–10.0 (T)) are shown in Fig. 4A. The results indicated that those values depend on the incident electron energy but are irrespective of the magnetic flux density. In the range of incident electron energy from 0.1 to 300 keV, there is no SMF impact on *Y*_DSB_ for various intensities of magnetic field in both parallel and perpendicular orientations. The DNA damage simulation based on *etsmode* suggests that the SMF effects do not appear at the DNA (nanometer) scale.

When irradiating high-energy electrons in liquid water, numerous secondary electrons with several tens of eV and Auger electrons with about 500 eV from the inner shells are generated. To illustrate the contribution of secondary electrons to DSB induction, we also estimated *Y*_DSB_ without considering the secondary electrons or Auger electrons. The result is shown in Fig. 4B. Focusing on 100-keV electrons, the *Y*_DSB_ value without any secondary electrons including Auger electrons (red circles and line) becomes lower than that with all secondary electrons (blue diamonds and line). Furthermore, the *Y*_DSB_ value without any secondary electrons is almost zero. The maximum value of *Y*_DSB_ without any secondary electrons is 1.51 in the case of 0.4-keV electrons, which is about half the value for *Y*_DSB_ = 3.35 when considering all secondary electrons. These results affirm that the secondary electrons including Auger electrons are major contributors to induce DSBs for high energy electrons (over 100 keV)^28^. Low-energy secondary electrons can be produced by inelastic interactions within a few fsec, and the corresponding penetration length is approximately 10 nm. Also, as shown in Fig. 3, the gyration time and radius to induce SMF impact on the secondary electrons are more than several ps and several µm, respectively. The secondary electrons therefore slow down before being considerably affected by a magnetic field.

**Figure 5:**
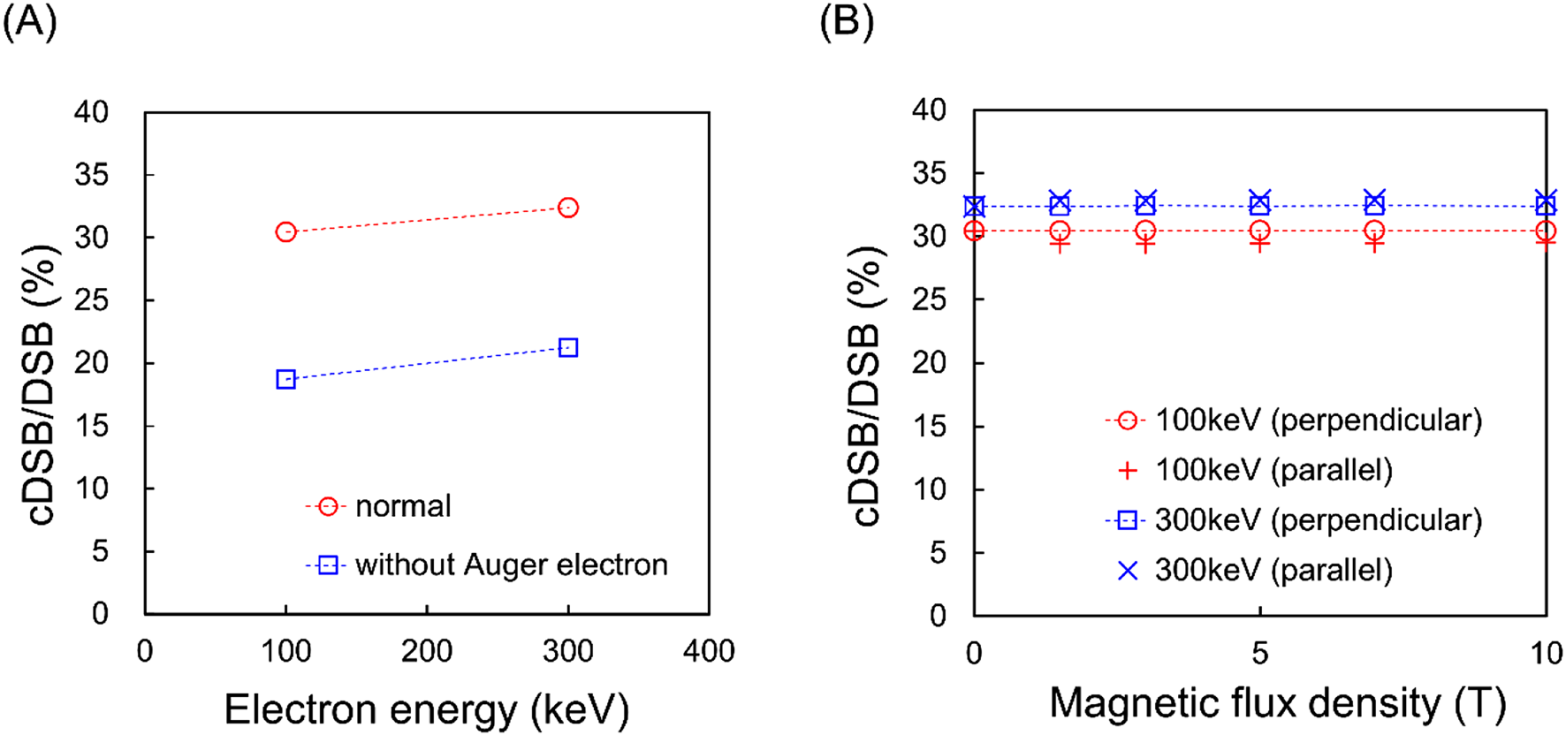
Various calculation results related complex DNA damage induction. (**A**) shows the relationship between the ratio of complex DSB yields per all DSBs yields (cDSB/DSB) and electron energy (100 and 300 keV). (**B**) shows the cDSB/DSB for 100, 300 keV electrons for various intensities of SMF, which is the ratio of the complex DSB yields calculated using PHITS (v. 3.27) and the analytical code for estimating DNA damage yields^24^.

Figure 5A shows that the ratio of DNA damage complexity (cDSB/DSB) for both electron energies decreases in the case of no Auger electrons. In addition, the ratios at 100 and 300 keV electrons for various intensities of the SMF is shown in Fig. 5B. The ratios were unchanged in any strength of magnetic field. This suggests that the content of complex lesions is independent of the intensity of the SMF. These results are corelated with the fact that Auger electrons contribute to DNA damage complexity and the energy of electrons is not enough to be affected by the SMF (Fig. 3).

The present estimation for DNA damage yields based on physical processes suggests no significant biological impacts caused by SMFs. This may be useful for interpreting the experimental data on surviving fractions after X-ray irradiation in the SMF^9–11^. However, some reports suggest the cell-killing effects for low-LET radiation increases in the direction parallel to the SMF^7,8^. To address the impact on cell-killing effects, we also calculated dose-mean lineal energy *y*_*D*_ (keV/μm)^36^ for site diameter *ϕ* = 1 μm, because a microdosimetric quantity has been related to cell survival probability^37,38^ as shown in Fig. S5 (supplementary material). The results show that energy deposition at sub-cellular scale is not affected by magnetic fields. As complex DNA damage is induced mainly by low-energy (keV and sub-keV) electrons, which are largely unaffected by SMF, the presence of SMF does not vary the ratio of complex DNA damage.

The *y*_D_ and *Y*_DSB_ values calculated in this study were only based on the physical interaction of electrons with liquid water as a substitute for biological tissues. This means that the simulation was conducted only for the direct effects (ionization and electronic excitation events). We did not consider the behavior of radical species such as their diffusion and mutual reactions in SMF. Future simulations shall address the effect of SMF on the chemical or biological phases. Unfortunately, there are no experimental data to compare with the yields of DNA damage calculated in this study.

## Conclusion

This work investigated the dose distributions, electron ranges and the early DNA damage yields in magnetic fields by means of PHITS track-structure simulations. In macro scale evaluations, the projected range and the dose distribution for therapeutic high-energy electrons (MeV order) can be modulated by magnetic fields. However, in DNA-scale evaluations, early DNA damage yields in magnetic fields were found to be independent of the SMF intensity. These simulations suggest that the treatment planning for MRgRT can be made in consideration of both changes of dose distribution and unaffected biological impacts. Since the present simulation was performed only based on physical processes, in the future it will be necessary to investigate chemical processes and the subsequent complex biological processes.

## Supplementary Information

Supplementary Information.

## Data Availability

All data generated or analysed during this study are included in this published article and its supplementary information files.

## References

[1] Lagendijk JJ (2008). MRI/linac integration. Radiother. Oncol..

[2] Raaymakers BW, Raaijmakers AJE, Kotte ANTJ, Jette D, Lagendijk JJW (2004). Integrating a MRI scanner with a 6 MV radiotherapy accelerator: Dose deposition in a transverse magnetic field. Phys. Med. Biol..

[3] Mutic S, Dempsey JF (2014). The ViewRay system: Magnetic resonance-guided and controlled radiotherapy. Semin. Radiat. Oncol..

[4] Raaijmakers AJ, Raaymakers BW, Lagendijk JJ (2005). Integrating a MRI scanner with a 6 MV radiotherapy accelerator: Dose increase at tissue-air interfaces in a lateral magnetic field due to returning electrons. Phys. Med. Biol..

[5] Nakahara T, Yaguchi H, Yoshida M, Miyakoshi J (2002). Effects of exposure of CHO-K1 cells to a 10-T static magnetic field. Radiology.

[6] Takatsuji T, Sasaki MS, Takekoshi H (1989). Effect of static magnetic field on the induction of chromosome aberrations by 4.9 MeV protons and 23 MeV alpha particles. J. Radiat. Res..

[7] Inaniwa T (2020). Effect of external magnetic fields on biological effectiveness of proton beams. Int. J. Radiat. Oncol. Biol. Phys..

[8] Inaniwa T (2019). Enhancement of biological effectiveness of carbon-ion beams by applying a longitudinal magnetic field. Int. J. Radiat. Biol..

[9] Rockwell S (1977). Influence of a 1400-gauss magnetic field on the radiosensitivity and recovery of EMT6 cells in vitro. Int. J. Radiat. Biol..

[10] Nath R, Schulz RJ, Bongiorni P (1980). Response of mammalian cells irradiated with 30 MV X-rays in the presence of a uniform 20-kilogauss magnetic field. Int. J. Radiat. Biol..

[11] Wang L (2016). Biological responses of human solid tumor cells to X-ray irradiation within a 1.5-Tesla magnetic field generated by a magnetic resonance imaging-linear accelerator. Bioelectromagnietics.

[12] Nikjoo H, Girard P (2012). A model of the cell nucleus for DNA damage calculations. Int. J. Radiat. Biol..

[13] Friedland W, Dingfelder M, Kundrát P, Jacob P (2011). Track structures, DNA targets and radiation effects in the biophysical Monte Carlo simulation code PARTRAC. Mutat. Res..

[14] Date H, Sutherland KL, Hasegawa H, Shimozua M (2007). Ionization and excitation collision processes of electrons in liquid water. Nucl. Instrum. Methods Phys. Res. B.

[15] Salvat, F. PENELOPE-2018: A code system for Monte Carlo simulation of electron and photon transport. NEA/MBDAV/R(2019)1. ISBN: 9789264489950 (2019).

[16] Sempau J, Acosta E, Baro J, Fernández-Varea JM, Salvat F (1997). An algorithm for Monte Carlo simulation of coupled electron-photon transport. Nucl. Instrum. Methods Phys. Res. B.

[17] Incerti S (2010). The Geant4-DNA project. Int. J. Model. Simul. Sci. Comput..

[18] Schuemann J (2019). TOPAS-nBio: An extension to the TOPAS simulation toolkit for cellular and sub-cellular radiobiology. Radiat. Res..

[19] Kirkby C, Stanescu T, Fallone BG (2009). Magnetic field effects on the energy deposition spectra of MV photon radiation. Phys. Med. Biol..

[20] Bug MU (2010). Effect of a magnetic field on the track structure of low-energy electrons: A Monte Carlo study. Eur. Phys. J. D.

[21] Sato T (2018). Features of particle and heavy ion transport code system (PHITS) version 3.02. J. Nucl. Sci. Technol..

[22] Kai T, Yokoya A, Ukai M, Watanabe R (2015). Cross sections, stopping powers, and energy loss rates for rotational and phonon excitation processes in liquid water by electron impact. Radiat. Phys. Chem..

[23] Kai T, Yokoya A, Ukai M, Fujii K, Watanabe R (2015). Thermal equilibrium and prehydration processes of electrons injected into liquid water calculated by dynamic Monte Carlo method. Radiat. Phys. Chem..

[24] Matsuya Y (2020). A simplified cluster analysis of electron track structure for estimating complex DNA damage yields. Int. J. Mol. Sci..

[25] Matsuya Y (2022). Track-structure mode in particle and heavy ion transport code system (PHITS): Application to radiobiological research. Int. J. Radiat. Biol..

[26] Matsuya Y (2021). Verification of KURBUC-based ion track structure mode for proton and carbon ions in the PHITS code. Phys Med Biol..

[27] Hirayama, H., Namito, Y., Bielajew, A. F., Wilderman, S. J. & Nelson, W. R. The EGS5 code system. SLAC Report 730, prepared for the Department of Energy, USA (2005).

[28] Nikjoo H, Oneill P, Goodhead DT, Terrissol M (1997). Computational modelling of low-energy electron-induced DNA damage by early physical and chemical events. Int. J. Radiat. Biol..

[29] Iwamoto Y (2017). Benchmark study of the recent version of the PHITS code. J. Nucl. Sci. Technol..

[30] Francis Z (2011). Molecular scale track structure simulations in liquid water using the Geant4-DNA Monte-Carlo processes. Appl. Radiat. Isot..

[31] Friedland W, Jacob P, Paretzke HG, Stork T (1998). Monte Carlo simulation of the production of short DNA fragments by low-linear energy transfer radiation using higher-order DNA models. Radiat. Res..

[32] Botchway SW, Stevens DL, Hill MA, Jenner TJ, O’Neill P (1997). Induction and rejoining of dna double-strand breaks in Chinese hamster V79–4 cells irradiated with characteristic aluminum K and copper L ultrasoft X rays. Radiat. Res..

[33] Folkard M (1993). Measurement of DNA damage by electron with energies between 25 and 4000 eV. Int. J. Radiat. Biol..

[34] de Lara CM, Hill MA, Jenner TJ, Papworth D, O’Neill P (2001). Dependence of the yield of DNA double-strand breaks in Chinese hamster V79–4 cells on the photon energy of ultrasoft X rays. Radiat. Res..

[35] Fulford J, Nikjoo H, Goodhead DT, O’Neill P (2001). Yields of SSB and DSB induced in DNA by Al_K_ ultrasoft X-rays and α-particles: Comparison of experimental and simulated yields. Int. J. Radiat. Biol..

[36] ICRU. Microdosimetry. Report 36. International Commission on Radiation Units and Measurements. Bethesda: MD (1983).

[37] Matsuya Y, Fukunaga H, Omura M, Date H (2020). A model for estimating dose-rate effects on cell-killing of human melanoma after boron neutron capture therapy. Cells.

[38] Parisi A (2020). Development of a new microdosimetric biological weighting function for the RBE_10_ assessment in case of the V79 cell line exposed to ions from ^1^H to ^238^U. Phys. Med. Biol..

