# Author Correction: Impact of the Lorentz force on electron track structure and early DNA damage yields in magnetic resonance-guided radiotherapy

**DOI:** 10.1038/s41598-023-29348-8

**Published:** 2023-02-08

**Authors:** Yoshie Yachi, Takeshi Kai, Yusuke Matsuya, Yuho Hirata, Yuji Yoshii, Hiroyuki Date

**Affiliations:** 1 Graduate School of Health Sciences, Hokkaido University, Kita-12 Nishi-5, Kita-ku, Sapporo, Hokkaido 060-0812 Japan; 2 Nuclear Science and Engineering Centre, Research Group for Radiation Transport Analysis, Japan Atomic Energy Agency (JAEA), 2-4 Shirakata, Tokai, Ibaraki 319-1195 Japan; 3 Central Institute of Isotope Science, Hokkaido University, Kita-15 Nishi-7, Kita-ku, Sapporo, Hokkaido 060-0815 Japan; 4 Faculty of Health Sciences, Hokkaido University, Kita-12 Nishi-5, Kita-ku, Sapporo, Hokkaido 060-0812 Japan

Correction to: *Scientific Reports* https://doi.org/10.1038/s41598-022-18138-3, published online 30 September 2022

The original version of this Article contained an error in Figure 2, where the y-axis label “Range mm” in panel (A) was incorrectly described as “Range nm”, and the y-axis units in panel (B) “2.5”, “2.0”, “1.5”, “1.0”, “0.5” and “0.0” were incorrectly described as “25”, “20”, “15”, “10”, “5” and “0”, respectively. The original Figure 2 and accompanying legend appear below.

**Figure 2:**
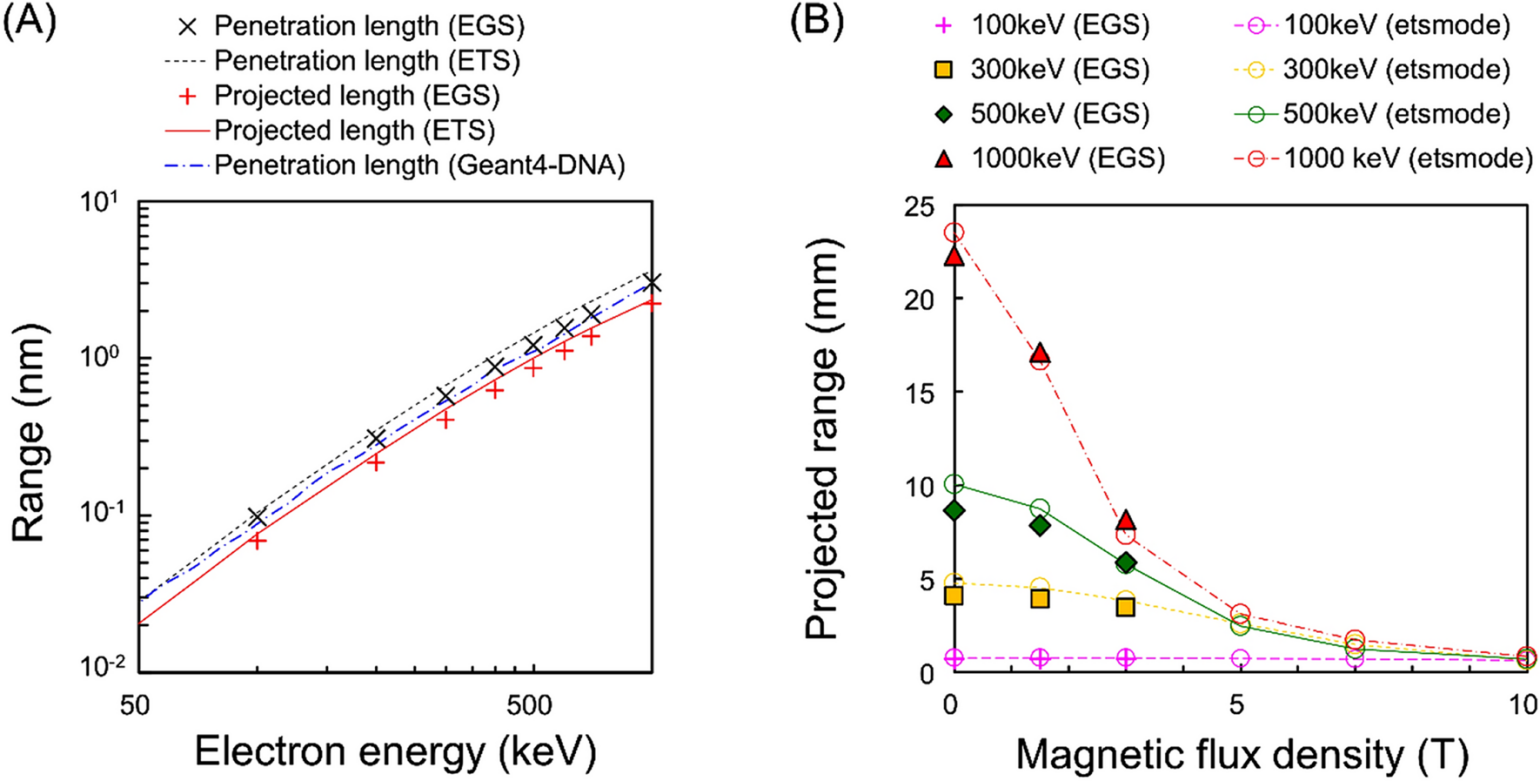
Electron ranges in SMFs calculated by *etsmode* and EGS. (**A**) shows the penetration length and projected range in the absence of a SMF (*B* = 0.0 T) and (**B**) shows the projected range in the presence of various SMF strengths (*B* = 0.0–10.0 T). The simulation results by *etsmode* and EGS are in good agreement with each other and other simulation results by the Geant4-DNA toolkit^30^. As shown in Fig. 2B, the projected range of high-energy electrons is largely affected by the SMF strength.

The original Article has been corrected.

