# Supplementary material for: Impact of the Lorentz force on electron track structure and early DNA damage yields in magnetic resonance-guided radiotherapy
Source: Sci Rep. 2022 Sep 30;12:16412. doi: 10.1038/s41598-022-18138-3 (PMC9525613; doi:10.1038/s41598-022-18138-3)
Supplement: Supplementary file 1 — Supplementary Information. [file 41598_2022_18138_MOESM1_ESM.docx]

***Supplementary data***

**Impact of the Lorentz Force on Electron Track Structure and Early DNA damage yields in Magnetic Resonance-Guided Radiotherapy**

Yoshie Yachi^1^, Takeshi Kai^2^, Yusuke Matsuya^2^, Yuho Hirata^2^, Yuji Yoshii^3,4^, Hiroyuki Date^4*^

This supplementary data includes seven figures: Figure S1. Electron track structure in parallel or perpendicular to SMFs; Figure S2. Comparison of dose distributions calculated by EGS and *etsmode*; Figure S3. Comparison of dose distribution for 10-MeV electrons in with no magnetic field; Figure S4. Dose distributions of electrons under 1-MeV in a SMF; Figure S5. Dose-mean lineal energy *y_D_* by the electrons under 1-MeV in a SMF.

**
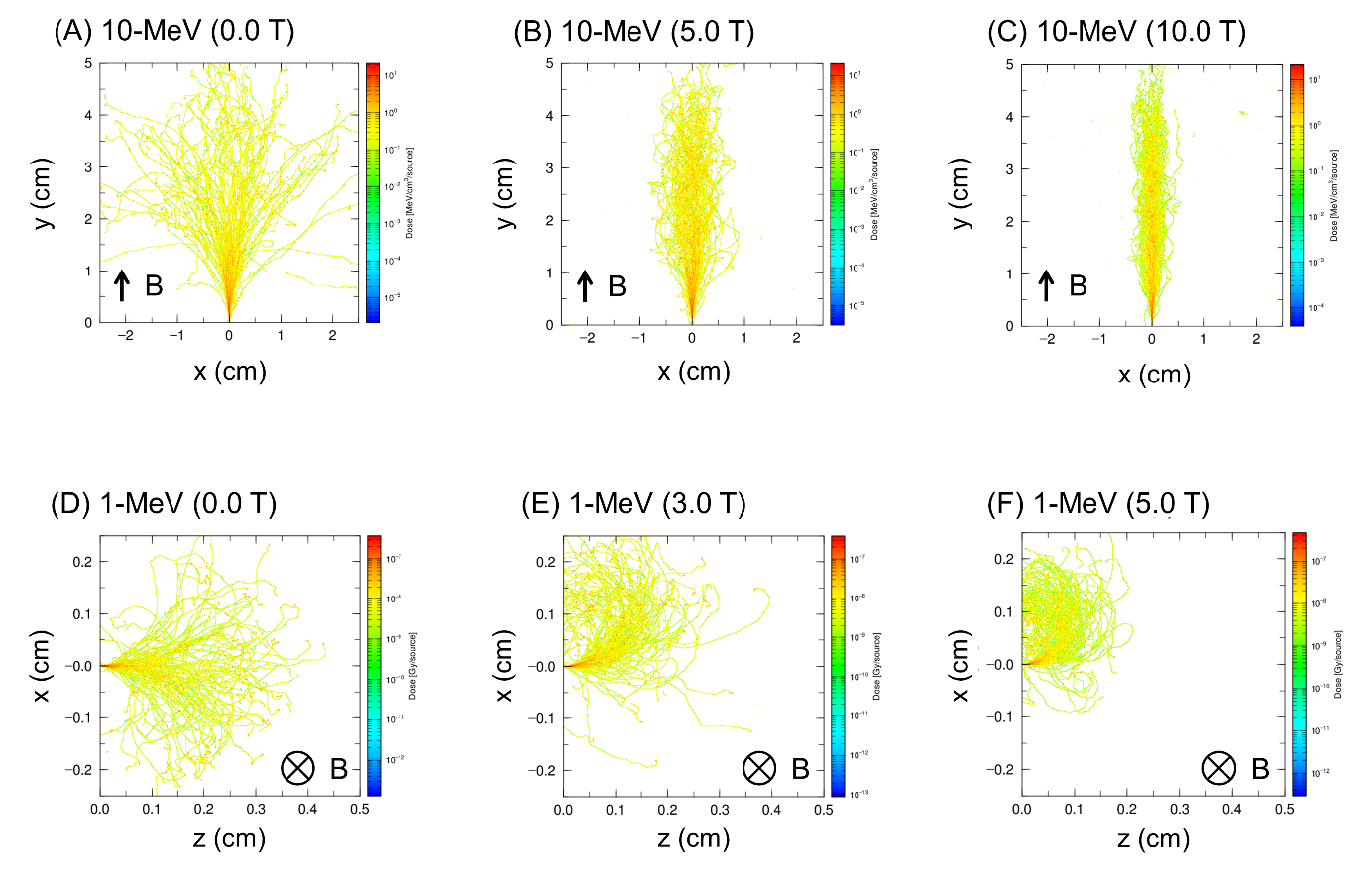
**

**Figure S1. Electron track structure in parallel or perpendicular to SMFs.** Track structures of electrons in SMFs were calculated by EGS. Electron energy is 10-MeV (for (A), (B), (C)) and 1-MeV (for (D), (E), (F)). Magnetic flux density, (A), (B), (C) are 0.0 T, 5.0 T, 10.0 T, and (D), (E), (F) are 0.0 T, 3.0 T and 5.0 T, respectively. For (A), (B), (C), incident electrons start at y=0 in the positive direction of y-axis and the SMF is in the y-axis direction (parallel to the direction of electron incident). For (D), (E), (F), incident electrons start at z=0 in the positive direction of z-axis and the SMF is in the y-axis direction (to the other side perpendicular to the paper).


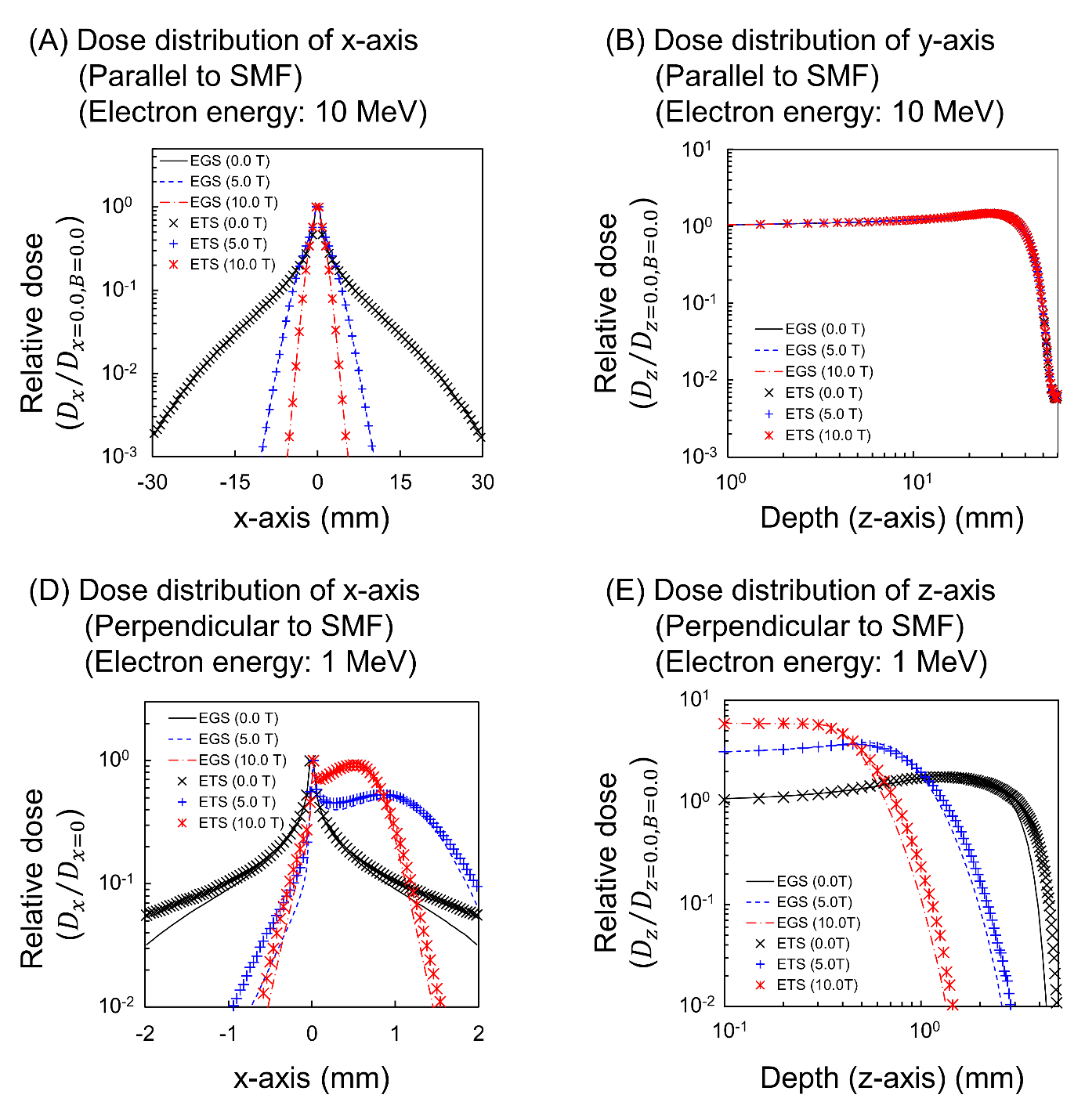


**Figure S2. Comparison of dose distributions calculated by EGS and *etsmode*.** Dose distributions along the x-axis (A) and the y-axis (B) for 10-MeV incident electrons in the y-axis positive direction parallel to a SMF. The distribution along the x-axis (C) and the z-axis (D) for 1-MeV incident electrons in the z-axis positive direction perpendicular to a SMF. These calculations are used by EGS and *etsmode*. From the comparison of the distributions calculated by EGS and *etsmode*, it was confirmed that the dose distributions calculated by *etsmode* were in good agreement with those by EGS.


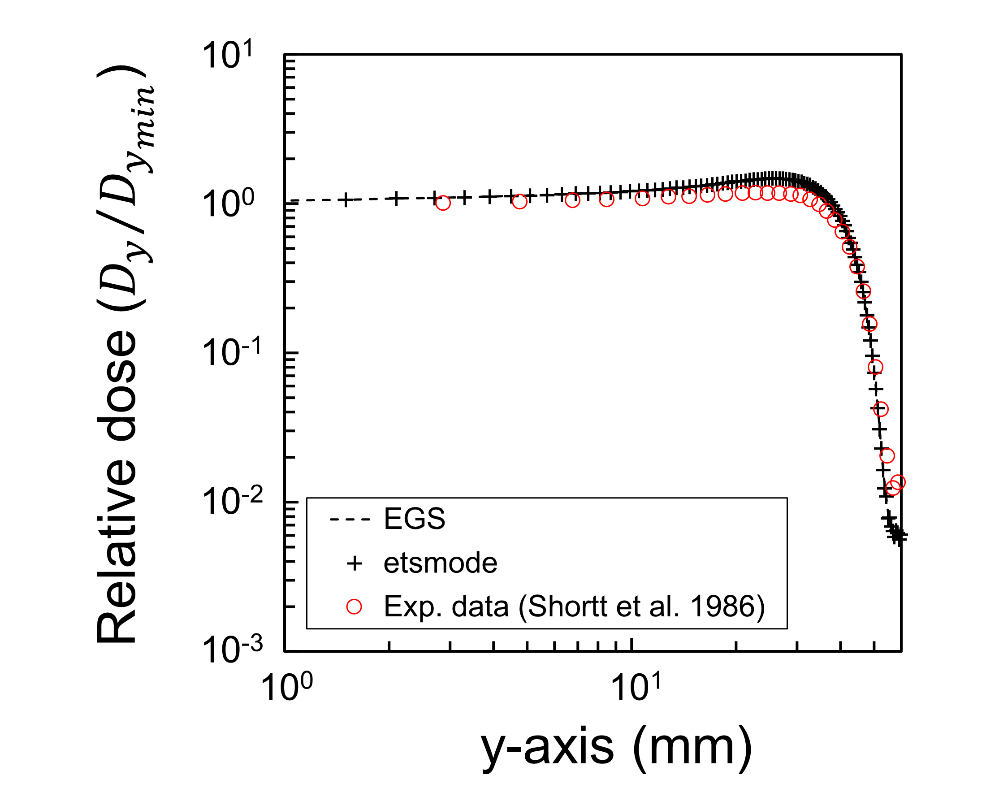


**Figure S3. Comparison of dose distribution for 10-MeV electrons in with no magnetic field.** The dose distributions as a function of depth for 10-MeV electrons in with no magnetic field were calculated by the EGS mode and *etsmode* (this study). The calculated distributions were compared with the experimental data reported by Shortt et al^1^. Both calculated distributions are in good agreement with the experimental data.

**
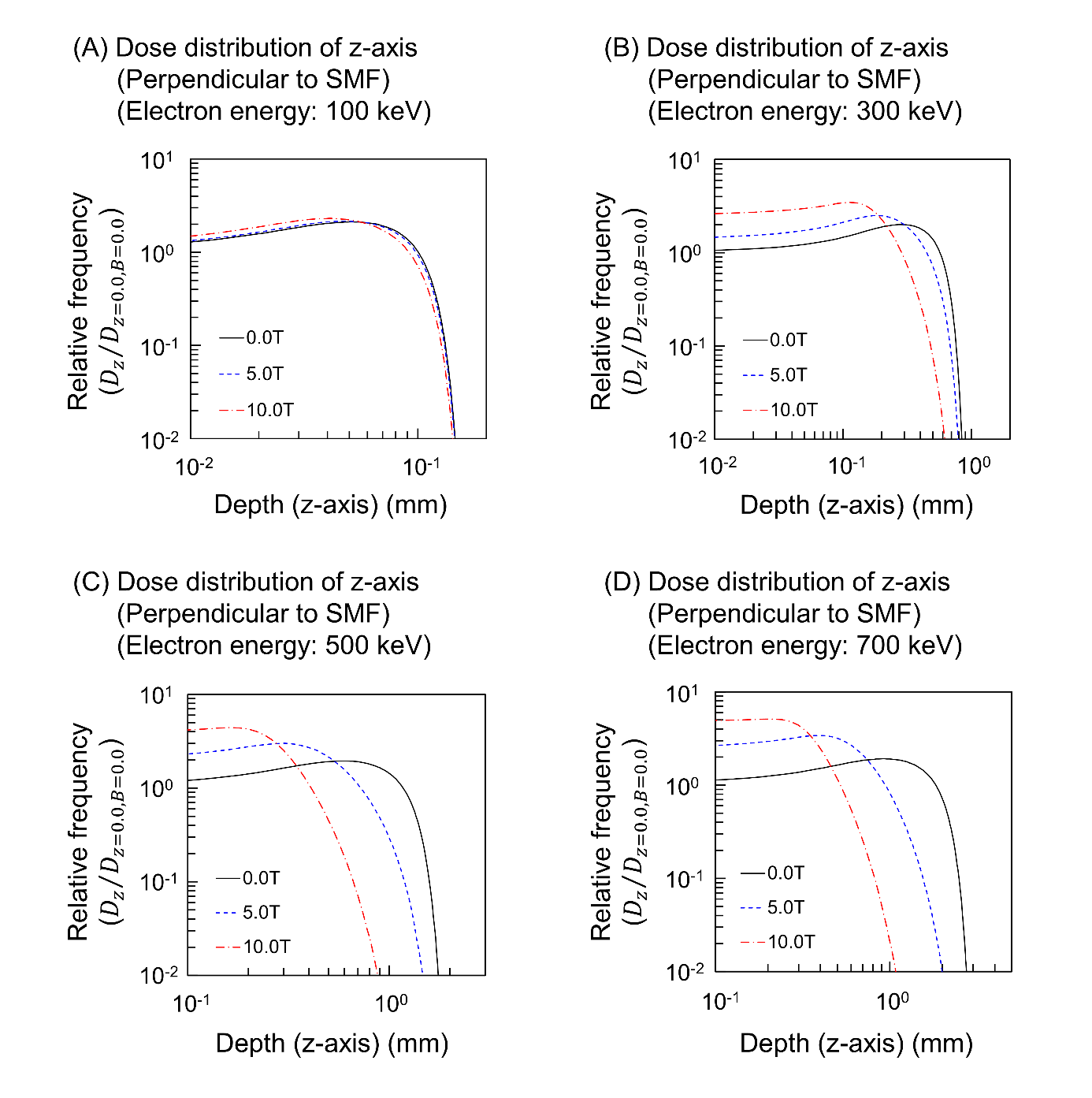
**

**Figure S4.** **Dose distributions of electrons under 1-MeV in a SMF.** Dose distributions along the z-axis for incident electrons with energy less than 1-MeV (100-700 keV) in the z-axis positive direction perpendicular to a SMF calculated by EGS. These distributions suggest that the impact of SMFs become smaller when the incident energy of electrons is lower, and the distribution of 100 keV electrons was almost unchanged by the SMF (up to 10.0 T).


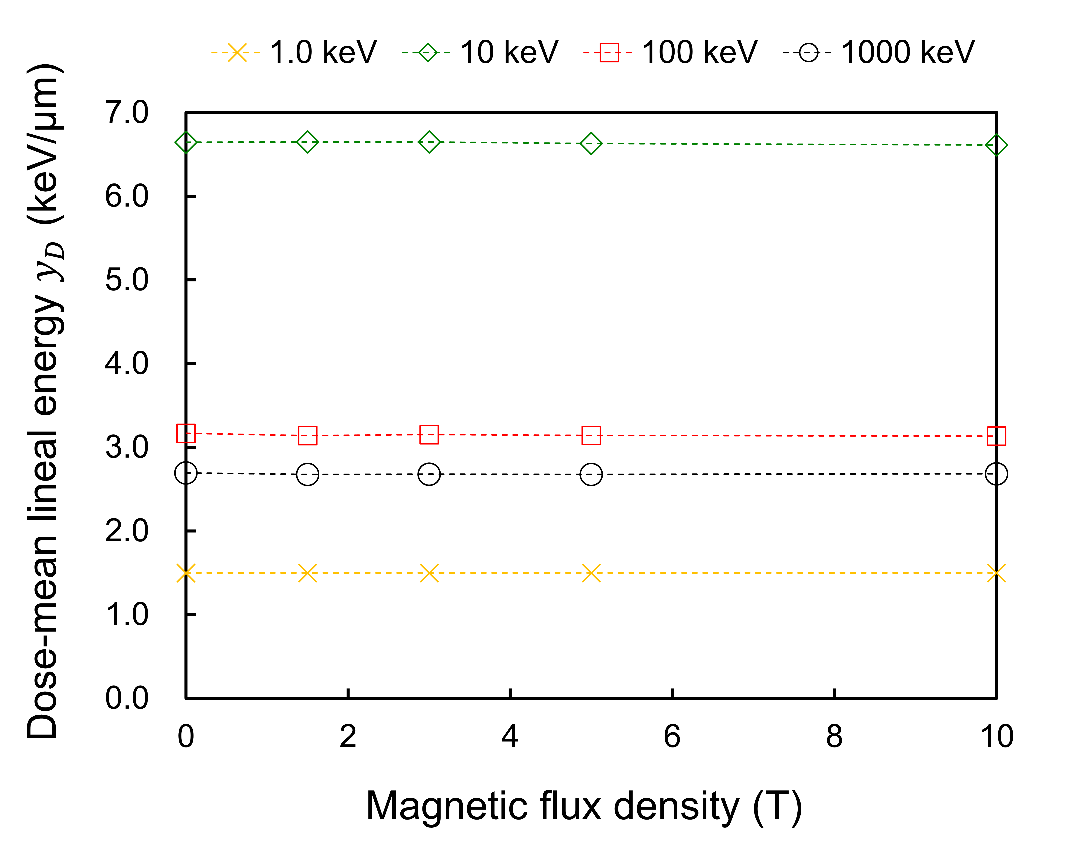


**Figure S5. Dose-mean lineal energy *y_D_* by electrons with energies under 1-MeV in a SMF.** Dose-mean lineal energy *y_D_* which is the microdosimetric quantity for various electron energies in the SMF (*B* = 0–10.0 T). The calculation of the *y_D_* value was performed by using a Monte Carlo code for electron transport, WLTrack^2^, which is the event-by-event track-structure simulation of each atomic interaction (full Monte-Carlo simulation). The site diameter was set to be 1.0 μm in this study. This calculation suggests that the *y_D_* value (which is related to cell survival probability) for 1-MeV electrons is unchanged by the SMF intensity *B* = 10.0 T.

**References**

1. Shortt, K. R., Ross, C. K., A F Bielajew, A. F. and Rogers, D. W. O. Electron beam dose distributions near standard inhomogeneities. *Phys. Med. Biol.* **3**(1): 235-249 (1986).
2. Date, H., Sutherland, K. L., Hasegawa, H. and Shimozua, M. Ionization and excitation collision processes of electrons in liquid water. *Nucl. Instrum. Methods Phys. Res. B* **265**, 515–520 (2007).
